# Factors that influence the implementation of AI-driven lifestyle monitoring in long-term care for older adults

**DOI:** 10.1093/geront/gnaf230

**Published:** 2025-10-03

**Authors:** Sjors Groeneveld, Tessa Dekkers, Lisette van Gemert-Pijnen, Ruud Verdaasdonk, Tijne Verveda, Robin Witteveen, Harmieke van Os-Medendorp, Marjolein den Ouden

**Affiliations:** Research Group Technology, Health & Care, School of Social Work, Saxion University of Applied Sciences, Enschede, The Netherlands; TechMed Center, Health Technology Implementation, University of Twente, Enschede, The Netherlands; Centre for eHealth and Wellbeing Research, Health and Technology, University of Twente, Enschede, The Netherlands; Centre for eHealth and Wellbeing Research, Health and Technology, University of Twente, Enschede, The Netherlands; TechMed Center, Health Technology Implementation, University of Twente, Enschede, The Netherlands; Centre for eHealth and Wellbeing Research, Health and Technology, University of Twente, Enschede, The Netherlands; Centre for eHealth and Wellbeing Research, Health and Technology, University of Twente, Enschede, The Netherlands; Faculty Health, Sports, and Social Work, Inholland University of Applied Sciences, Amsterdam, The Netherlands; Spaarne Gasthuis Academy, Haarlem, The Netherlands; Research Group Technology, Health & Care, School of Social Work, Saxion University of Applied Sciences, Enschede, The Netherlands; Research Group Care and Technology, Regional Community College of Twente, Hengelo, The Netherlands

**Keywords:** Ageing in place, Artificial intelligence, Monitoring, Implementation

## Abstract

**Background and Objectives:**

AI-driven lifestyle monitoring systems collect data from ambient, motion, contact, light, and physiological sensors placed in the home, enabling AI algorithms to identify daily routines and detect deviations to support older adults “aging in place.” Despite its potential to support several challenges in long-term care for older adults, implementation remains limited. This study explored the facilitators and barriers to implementing AI-driven lifestyle monitoring in long-term care for older adults, as perceived by formal and informal caregivers, as well as management, in both an adopting and nonadopting healthcare organization.

**Research Design and Methods:**

A qualitative interview study using semi-structured interviews was conducted with 22 participants (5 informal caregivers, 10 formal caregivers, and 7 participants in a management position) from two long-term care organizations. Reflexive thematic analysis, guided by the nonadoption, abandonment, scale-up, spread, and sustainability (NASSS) framework, structured findings into facilitators and barriers.

**Results:**

In all, 12 facilitators and 16 barriers were identified, highlighting AI-driven lifestyle monitoring as a valuable, patient-centered, and unobtrusive tool enhancing care efficiency and caregiver reassurance. However, barriers such as privacy concerns, notification overload, training needs, and organizational alignment must be addressed. Contextual factors, including regulations, partnerships, and financial considerations, further influence implementation.

**Discussion and Implications:**

This study showed that to optimize implementation of AI-driven lifestyle monitoring, organizations should address privacy concerns, provide training, engage in system (re)design, and create a shared vision. A comprehensive multi-level approach across all levels is essential for successful AI integration in long-term care for older adults.

##  

Long-term care is facing substantial challenges due to significant demographic and systemic shifts. Increased life expectancy (United Nations Department of Economic and Social Affairs, 2022) is driving greater demand for care (Prince et al., 2015). Yet a shortage of qualified healthcare professionals is adding further pressure to the system (Peters, 2023). This brings significant challenges, including rising healthcare costs but also increased pressure on healthcare professionals and informal caregivers (Daalhuizen et al., 2018). Government policies responding to these challenges encourage self-reliance and resilience by supporting older adults in seeking help from social networks first and arranging formal care themselves when needed (Dutch Ministry of Health, 2018). A key part of this approach is promoting “aging in place,” i.e., older adults living independently at home for as long as possible (Fritz & Dermody, 2019; Kivimäki et al., 2020). This is considered a cost-effective option compared to institutional care (Daalhuizen et al., 2018). Many older adults also prefer to remain in their own homes as they age, rather than moving to institutionalized care (CBS, 2020). While this shift in care delivery presents opportunities for the long-term care system, it also highlights the need for effective solutions to ensure that older adults can live comfortably and securely at home (Liu et al., 2019).

One of these possible solutions is the use of innovation and technologies that can support older adults to age in place. Several technological innovations are being developed, and one of these promising developments is the use of artificial intelligence(AI) (Badawy & Shaban, 2025). AI functions by simulating human intelligence in machines, allowing for problem-solving and learning capabilities that mimic human cognition (Shang, 2021).

In long-term care, AI can serve various functions, including monitoring the health and daily activities of older adults in smart home settings (Dermody & Fritz, 2019). This type of technology, known as AI-driven lifestyle monitoring (Groeneveld et al., 2024), is used to obtain insights into an individual’s behavior, such as daily routines, habits, and activity patterns (Naccarelli et al., 2022; Wrede et al., 2021). AI-driven lifestyle monitoring systems (see Figure 1) operate by collecting input from ambient and environmental sensors placed throughout the home, including motion, contact, light sensors (Fritz & Dermody, 2019), and physiological sensors (Krizea et al., 2022). These sensors continuously track the home environment and the older adult living there, feeding data to an AI algorithm that learns daily routines, habits, and activity patterns and identifies deviations therein (Dawadi et al., 2013). Detected deviations may include disruptions in sleep patterns, increased bathroom visits, reduced activity levels (Fritz & Dermody, 2019), and even emotional changes (Vuijk et al., 2023). When a deviation is identified, the system notifies users, including formal and informal caregivers (Le et al., 2014), providing recommendations to facilitate timely preventive actions and early interventions (Sapci & Sapci, 2019).

**Figure 1.**
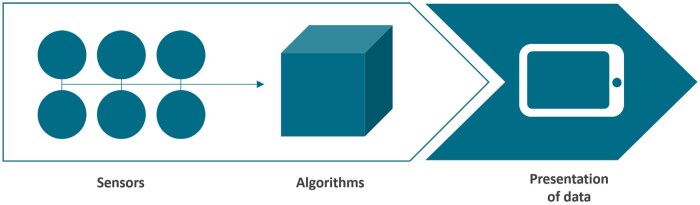
Visualization of an AI-driven lifestyle monitoring system. Diagram showing a data flow from left to right: six sensor icons feeding into a cube labelled “Algorithms,” which processes the data and outputs it to a tablet icon representing “Presentation of data.”

## Implementation challenges

Understanding the factors that influence implementation of AI-driven lifestyle monitoring requires balancing the diverse perspectives of multiple stakeholders, including informal caregivers who want more supervision of their loved one’s situation (Wrede et al., 2021), formal caregivers wanting to deliver high-quality care (Groeneveld et al., 2023), and care organizations who need to adapt to a new model of long-term care (Dutch Ministry of Health, 2018). Aligning these goals is important to enhance the likelihood of effective implementation and utilization (Greenhalgh et al., 2017; Ho, 2020) of AI-driven lifestyle monitoring. In this study, we build upon previous research that has examined the attitudes of formal and informal caregivers toward non-AI lifestyle monitoring, which has shown that in-home monitoring can contribute to a shift from reactive to more proactive and less obtrusive care if aspects such as trust, reliability, and privacy and safety are taken into account (Sharma et al., 2023; Wrede et al., 2021). This study expands the focus of these previous studies by also examining implementation aspects at organizational and societal levels (Greenhalgh et al., 2017), incorporating the perspective of management to recognize their complementary views on factors that facilitate or hinder the successful implementation of AI-driven lifestyle monitoring in long-term care for older adults. Additionally, to provide a more comprehensive overview of barriers and facilitators that affect implementation, the study incorporates perspectives from both a long-term care organization that has already adopted AI-driven lifestyle monitoring and one that has not yet adopted the technology.

Therefore, the aim of this study is to identify the facilitators and barriers that formal and informal caregivers as well as management perceive regarding the implementation of AI-driven lifestyle monitoring systems in long-term care for older adults.

## Methods

### Study design

Between February and September 2024, we conducted a qualitative interview study to identify the facilitators and barriers that formal and informal caregivers, as well as management, perceive regarding the implementation of AI-driven lifestyle monitoring systems in long-term care for older adults. This qualitative method was chosen due to its effectiveness in exploring diverse stakeholder perspectives in depth (Pyo et al., 2023). The consolidated criteria for reporting qualitative studies (COREQ) checklist was used for reporting this study (Tong et al., 2007).

#### Study setting

This study was conducted at two long-term care organizations in the Netherlands, both specializing in care for older adults. The services of the two involved long-term care organizations include, among others, home care to support aging in place and residential care. Since this study focuses on the implementation of AI-driven lifestyle monitoring to support aging in place, the emphasis is specifically on home care services. The approach to include two organizations captures both the practical lessons learned through implementation (by the adopting organization) and the unmet needs or uncertainties that hinder implementation (by the nonadopting organization), thereby offering recommendations for organizations at different stages of the implementation process.

The adopting organization is characterized by its forward-looking strategy of embracing technological innovation as a means to provide more efficient and personalized care. With nearly 4,000 staff members and around 1,000 volunteers, it serves over 12,000 clients. The organization has actively implemented AI-driven lifestyle monitoring within its home care services (in various pilot projects) as part of its broader strategy to enhance independence and quality of life for older adults living at home.

The nonadopting organization emphasizes a community-oriented, personalized care approach, prioritizing self-management and close collaboration with informal caregivers and volunteers. It operates with approximately 2,800 staff members and 1,000 volunteers, focusing on both home care and residential care. Although it has not yet adopted AI-driven monitoring, it is exploring the potential of care technologies to support older adults in maintaining autonomy at home.

The interviews were conducted by two graduate researchers (R.W. and T.J.V.) who had received training in qualitative research methods. Both researchers were employees of the participating long-term care organizations during the study. Importantly, neither researcher was directly involved in the implementation or use of AI-driven lifestyle monitoring within their organizations. To enhance credibility two other researchers (S.W.M.G. and T.D.) supervised the data collection and data analysis.

#### Participants

Interviews were carried out with informal caregivers, formal caregivers, and management at the two long-term care organizations. Participants had to be over 18 years old, able to understand Dutch, and be employed as formal caregiver or in a management role related to the use of technology or serving as an informal caregiver (often family members) of a patient receiving care at one of the two long-term care organizations to be eligible for the study. The purposive sampling strategy was structured to ensure the inclusion of diverse perspectives across stakeholder groups, targeting individuals with varying roles, levels of responsibility, and exposure to technology within the care organizations. Specifically, recruitment aimed to include those involved in decision-making about technology use (experts, advisors, or those in a management position), direct care providers (for example, nurses), and informal caregivers (often family members). Following this initial purposive sampling phase, snowball sampling was employed to identify additional participants, as suggested by interviewees (Parker & Geddes, 2019). At the end of each interview, participants were asked to suggest other relevant individuals, who were then invited to participate if they met the inclusion criteria. Participants were contacted via email, informing them about the purpose of the study and the interviewers’ interest in the research topic. After agreeing to participate, they received an informational letter detailing the research and interview expectations. All participants signed an informed consent form before the interview started.

#### Theoretical framework

A reflexive thematic analysis with a deductive approach was used based on the nonadoption, abandonment, scale-up, spread, and sustainability (NASSS) framework (Braun & Clarke, 2021; Greenhalgh et al., 2017). This framework is commonly used for implementation research in healthcare as it recognizes the complex, multidimensional challenges of technology implementation, including the dynamic interactions across individual, organizational, and societal levels. See Table 1 for the seven domains of the NASSS framework that were used to categorize the interview guide questions and structure the results.

**Table 1.** Domains of Nonadoption, Abandonment, Scale-up, Spread, and Sustainability (NASSS) Framework (Greenhalgh et al., 2017).

Domain	Focus
**Condition**	Focuses on the condition that a technology aims to address.
**Technology**	Focuses on the simplicity, stability, and usability of a technology.
**Value proposition**	Focuses on the added value a technology provides to all stakeholders.
**Adopters**	Focuses on the users of a technology, including their willingness and ability to adopt it.
**Organization**	Focuses on how well a technology aligns with the organizational culture, structure, and resources.
**Wider system**	Focuses on external influences such as legislation, policy, and economic factors that impact adoption.
**Embedding and adaptation over time**	Focuses on how a technology is integrated into practice and its ability to adapt to future change.

**Table 2.** Overview of participants.

Participant type	Role(s)	Description	*N*
**Informal caregivers**	Informal caregiver	Informal caregivers, often family members of patients, assist older adults in maintaining independent living at home.	5
**Formal caregivers**	Nurses	Nurses are responsible for providing personalized support and medical care to patients. Job titles include nurse (assistant), vocational nurse, and coordinating district nurse.	7
	Dementia case manager	Dementia case managers support dementia patients and their families to ensure that the provided care is tailored to their specific needs.	2
	Coordinator long-term home care team	A coordinator of a long-term home care team oversees and organizes care services for patients while managing team operations and compliance with care standards.	1
**Management**	Healthcare technology expert	Healthcare technology experts are responsible for implementing and integrating technology within the organization and training staff.	1
	Business process advisor	A business process advisor is responsible for improving and optimizing business processes, developing and implementing organizational changes and managing related projects.	1
	ICT advisor	An ICT advisor is responsible for maintaining and optimizing the organization’s ICT infrastructure. This role involves close collaboration with the healthcare technology experts to ensure seamless integration and functionality of healthcare technologies.	1
	Director of home care	A director of home care supervises all aspects of home care within the organization.	1
	Innovation manager	An innovation team is responsible for innovation and healthcare technologies within the organization. The team is managed by its innovation manager who is responsible for managing the team and making sure that the organization innovates its care to adapt to patient, employee and wider care system needs.	1
	Innovation project leader	An innovation project leader is part of the innovation team and is responsible for leading innovation projects, like the implementation of AI-driven lifestyle monitoring, within the organization.	1
	Transformation manager	A transformation manager plays a role in successfully implementing large-scale changes within the organization and ensuring that the organizations strategic objectives are met.	1

### Data collection and analysis

#### Data collection

To encourage candid responses and minimize the risk of participants influencing each other’s opinions (particularly the risk of withholding criticism in group settings), we conducted one-on-one interviews (lasting 50 min on average) with informal caregivers, formal caregivers, and management.

All interviews were semi-structured and guided by interview guides, which were developed for the different participant groups: informal caregivers, formal caregivers, and management. These guides, which were pilot tested in the research team, were structured according to the NASSS framework (Greenhalgh et al., 2017) and contained domain-specific questions designed to gather relevant insights based on the participant’s role. For instance, informal caregivers and formal caregivers primarily addressed questions related to adopters, while management focused on organization and the wider system. This approach ensured the interviews remained focused and enabled meaningful insights across participant responses (Jamshed, 2014). Interviews were conducted either online via Teams or in person, depending on the participant’s preference. The interviews were recorded using Teams (online) or a voice recorder (in-person) for analysis purposes.

#### Data analysis

For the analysis of the interviews, recordings were first transcribed into text and carefully reviewed for accuracy. A reflexive thematic analysis (Braun & Clarke, 2021) with a deductive approach was conducted. Initial codes were generated based on the transcript by two primary coders (R.W. and T.J.V.), who independently coded the data, followed by collating codes into potential themes. This process was further supported by two additional researchers (S.W.M.G. and T.D.), who reviewed the coded data to provide additional perspectives and ensure consistency. This concluded the first round of coding, which importantly did not yet include the definition of themes as facilitators or barriers. After completing this initial coding and identifying potential themes, all authors discussed the interpretations to reach consensus on defining and naming barriers and facilitators. Differences in coding were few. When these occurred, they were necessary to more clearly indicate whether a theme reflected either a facilitator or a barrier. For example, an earlier version of the final theme “negative experiences with costumer service” was named simply “customer service” and did not yet reflect that specifically negative experiences contributed to this barrier. All differences in coding of this kind were resolved through discussion with the first two authors (S.W.M.G. and T.D.) and checked by the two primary coders (R.W. and T.J.V.). This multi-coder approach was employed to strengthen the reliability and validity of the findings. The analysis was performed using ATLAS.ti (version 24.1.1).

During analysis, findings were structured using a coding structure of domains and underlying themes. Each domain corresponds to the NASSS framework, while the themes represent the points raised by participants during the interviews. Themes were further clustered as facilitators and barriers and reported in tables alongside their definitions with representative participant quotes and a specification whether it was observed in the adopter and/or nonadopter organization and by which participant type (informal caregiver, formal caregiver, and/or management), to increase the reliability and validity of the interpretations (Braun & Clarke, 2021).

#### Member check

To further enhance the credibility of the interview data, a member check was conducted with participants (Erdmann & Potthoff, 2023). A detailed summary of the interview results was emailed to participants, with a request for comments or feedback on the findings. This allowed participants to verify the accuracy of the data and validate the interpretations. Furthermore, the member check provided an opportunity to address any misunderstandings or discrepancies, further strengthening the credibility of the collected data. Some participants gave additional insights during the member check, such as clarification on discussed topics, ensuring the reliability of the results.

#### Ethical approval

The Ethics Committee of the University of Twente (Behavioral, Management, and Social Sciences) provided ethical approval for this study (request number 240201). This research did not fall under the Dutch Medical Research Act (WMO) as it did not require participants to carry out specific actions or adopt particular behaviors. Anonymity and confidentiality were guaranteed to participants and they were informed of the voluntary involvement and the possibility to withdraw at any time.

## Results

### Participants

A total of 22 participants took part in this study, comprising of 5 informal caregivers, 10 formal caregivers (7 nurses, 2 dementia case managers, and 1 coordinator of a long-term home care team) and 7 participants in a management position (1 healthcare technology expert, 1 business process advisor, 1 ICT advisor, 1 director of home care, 1 innovation manager, 1 innovation project leader, and 1 transformation manager). The participants’ ages ranged from 20 to 70 years, including 17 women and 5 men, see Table 2 for an overview of participant types.

### Overview of themes

We identified 28 themes across all 7 domains of the NASSS framework (Greenhalgh et al., 2017), see Table 3. These included 12 facilitators (Table 4) that may improve the implementation of AI-driven lifestyle monitoring and 16 barriers (Table 5) that may negatively impact implementation. For each identified theme, it is specified whether it was observed in the adopter or nonadopter organization and which participant type (informal caregiver, formal caregiver, or management) identified it. Both facilitators and barriers were most likely to be discussed in relation to the value proposition domain, while the organizational domain was only discussed in relation to barriers and the adaptation over time domain only in relation to facilitators.

**Table 3.** Overview of Themes (Facilitators and Barriers), Structured along the Nonadoption, Abandonment, Scale-up, Spread, and Sustainability (NASSS) Framework Domains (Greenhalgh et al., 2017).

		Organization type (adopter/nonadopter)		Participant type (informal, formal, management)		
	Domain/theme	A	NA	IC	FC	M
1. Condition						
	Versatility (F)	✓	✓	✓	✓	✓
	Patients’ limited insight into condition (B)	✓			✓	✓
2. Technology						
	Integrated output (F)	✓	✓	✓	✓	✓
	Unobtrusiveness (F)	✓	✓	✓	✓	✓
	Lack of reliability (B)	✓	✓	✓	✓	✓
	Negative experience with customer service (B)	✓		✓	✓	✓
3. Value proposition						
	Reassurance (F)	✓	✓	✓	✓	✓
	Tailored care (F)	✓	✓	✓	✓	✓
	Care efficiency through prevention (F)	✓	✓	✓	✓	✓
	Invasion of privacy (B)	✓	✓	✓	✓	✓
	Potential for overtreatment (B)	✓		✓	✓	✓
	Strenuous feelings (B)	✓	✓	✓	✓	✓
	Price (B)	✓			✓	✓
4. Adopters						
	Patient-aligned (F)	✓	✓	✓	✓	✓
	Unclear actor upon notifications (B)	✓	✓	✓	✓	✓
	Training needed to use the system (B)	✓		✓	✓	✓
	Change in job priority (B)	✓	✓	✓	✓	✓
	Notification overload (B)	✓		✓	✓	✓
5. Organization						
	Lack of capacity (B)	✓			✓	✓
	Behavioral change in technology use is needed (B)	✓				✓
	Support deficiency (B)	✓			✓	
6. Wider system						
	Partnerships (F)	✓			✓	✓
	National regulations and priorities (F)	✓	✓	✓	✓	✓
	Sharing of data (F)	✓			✓	✓
	Lack of reimbursement (B)	✓	✓	✓	✓	✓
	Prevention costs and benefits are not aligned (B)	✓	✓		✓	✓
7. Embedding and adaptation over time						
	AI-driven lifestyle monitoring is future-proof (F)	✓	✓	✓	✓	✓
	Increasing complexity of extramural care (F)	✓	✓		✓	✓
						
F	Facilitator					
B	Barrier					
A	Adopter organization					
NA	Nonadopter organization					
IC	Informal caregivers					
FC	Formal caregivers					
M	Management					

**Table 4.** Overview of facilitators.

Domain/theme	Definition	Quote(s)
**1. Condition**	**The health-related factors and patient characteristics of users of AI-driven lifestyle monitoring.**	
Versatility	The use of AI-driven lifestyle monitoring is suitable for cognitive decline and other somatic conditions.	*“Right now, we mainly focus on dementia, but I believe we could adopt a much broader perspective. I think it could also be well substantiated for certain somatic conditions, especially when you consider the impact such conditions can have on someone’s autonomy. […]. It could help us assess patterns, identify deviations, and respond effectively, especially when individuals themselves may no longer have the ability to reflect on and communicate these changes.”—*Transformation manager
**2. Technology**	**The perceived features of the technology.**	
Integrated output	Users expected integrated observation displays, minimal notifications and adaptability to needs.	*“We shouldn’t have too many fragmented alarms; we should just capture everything in one.”—*Dementia case manager
Unobtrusiveness	The system must integrate seamlessly with the older adults’ environment.	*“I think it should be as unobtrusive as possible. People should know it’s there, but it shouldn’t be so obvious that they see strange devices on their shelves or walls and wonder what they are. If the design looks appealing, I think it will be easier to use.”—*Nurse *“Patients shouldn’t be bothered by it. Older adults with dementia can be suspicious, and if they see something that doesn’t seem right, it should be addressed properly. So, it’s best if the sensor is hidden.”* Business process advisor
**3. Value proposition**	**The overall value of the technology.**	
Reassurance	AI-driven lifestyle monitoring contributes to a sense of safety and provides reassurance.	*“My mother has fallen several times over the past few years. […] We have now decided not to rely solely on the nurses or family caregivers for monitoring but to also use a system. It’s for her own safety, and she appreciates it as well—it just gives her a sense of reassurance.”—*Informal caregiver *“It’s reassuring for me […], to have an extra safety net. At least, that’s how it feels to us—knowing there’s another tool in place to respond quickly if there’s an emergency.”—*Informal caregiver
Tailored care	The system enables care tailored to actual patient needs by collecting additional and objective data.	*“I think the positive value is that, if it were scaled up significantly, we could provide better care for many patients. This is because you would have a clearer understanding of their actual care needs and, in some cases, you might even prevent hospital admissions by addressing issues more quickly than you otherwise would have.”—*Project manager innovation *“Such technology [AI-driven lifestyle monitoring] can be very useful for saving time by quickly mapping out situations with accurate data. […] This allows you to provide the right care immediately, addressing issues before they escalate. By identifying problems early and offering appropriate care, you can prevent people from requiring costly interventions.”—*Dementia case manager (nonadopter)
Care efficiency through prevention	Care efficiency is expected to improve due to the preventive potential of AI-driven lifestyle monitoring.	*“Lifestyle monitoring can be very effective, especially if it prevents something that would otherwise require significant care, like a hospital visit for something preventable, such as delirium.”—*Innovation manager *“Twice now we have been able to detect a bladder infection early, for example. Which means you’re actually ahead of a lot of trouble, because we saw that someone was suddenly going to the toilet much more often.”—*Nurse
**4. Adopters**	**The adoption and continued use of the technology by different stakeholders.**	
Patient-aligned	AI-driven lifestyle monitoring is considered in line with the interests of the patient.	*“I think you just have to embrace it and focus on what’s best for the patient.”—*Informal caregiver
**5. Organization**	**The organization in which the technology has been implemented.**	
		
**6. Wider system**	**The wider regulatory and collaborative context around the current use of the technology.**	
Partnerships	Partnerships at the national, regional, and neighborhood levels positively impact the implementation of AI-driven lifestyle monitoring systems.	*“Well, [imagine this] Mr. Pieterse has wandered off or left the house. […] Everyone looks outside, helps him back home […] or watches over him until professional care arrives. If we could improve the system, living at home as someone prone to wandering could become much safer.”—*Innovation project leader *“Is that something a healthcare organization should actively promote within the community? Or is it something we should address together as a society perhaps?”—*ICT advisor
National regulations and priorities	National regulations and priorities, such as aging-in-place policies, support the use of the system.	*“[when discussing aging-in-place, red.] When I started in home care, we would sometimes see someone once a week with a wound on their leg, and that was it. Nowadays, they are discharged from the hospital and sent home with an IV [intravenous line]. The change has been enormous.”—*Nurse *“The desire comes from a sense of autonomy, self-reliance and individualism. If I can do it myself, then I want to do it myself.”—*Director of home care
Sharing of data	Data gathered by AI-driven lifestyle monitoring systems can be used in broader healthcare contexts beyond the care organization.	*“You are generating a lot of data and, based on that data, you can plan actions and interventions. That is nice to track within one organization, but if you combine it across everyone and can identify clearer patterns, I think you might even start to understand behavior better. Also, you could better map out, over periods like a year or a day, what behaviors and patterns you recognize. In the long term, you might even be able to design care pathways based on that.”—*Innovation project leader
**7. Embedding and adaptation over time**	**The long-term integration and use of the technology.**	
AI-driven lifestyle monitoring is future proof	Expectations of AI-driven lifestyle monitoring systems as future-proof within long term care.	*“We encourage more patient self-direction and self-reliance, and I definitely think that such an AI-driven lifestyle monitoring system, if developed further, can bring a lot towards patients living at home for longer. And so, with that in mind, I do think it’s leading up to future-proofing.”—*Nurse
Increasing complexity of extramural care	Anticipating longer stays at home for older adults, increasing complexity in care needs in times of nursing shortages are foreseen, which can be addressed by AI-driven lifestyle monitoring.	*“I think more older adults, who have to stay at home longer, will lead to increasingly complex care being provided at home. At the same time, the demand for care is increasing, while the number of caregivers is decreasing. So, we really have to look at how we’re going to organize things differently, and I think that’s really important.”—*Healthcare technology expert *“Well, we know that the dementia demographic is only going to increase and expand, so I believe this could be a very valuable addition, as it would provide us with a lot of valuable insights in the future.”—*Healthcare technology expert

**Table 5.** Overview of barriers.

Domain/theme	Definition	Quote(s)
**1. Condition**	**The health-related factors and patient characteristics of users of AI-driven lifestyle monitoring.**	
Patients’ limited insight into condition	Patients’ limited insight into their condition may deter the use of AI-driven lifestyle monitoring.	*“Our target group sometimes says, ‘Well, I don’t think I need it yet,’ and that’s not only due to suspicion but also a lack of insight into their illness. Of course, these things are connected, but sometimes it’s really just a lack of awareness about their condition: ‘Oh yeah, it sounds good, but not right now because I’m doing well at the moment’.”—*Dementia case manager
**2. Technology**	**The perceived features of the technology.**	
Lack of reliability	The reliability of AI-driven lifestyle monitoring, including potential errors, negatively affects its perceived trustworthiness.	*“If it does what it’s supposed to do, then it is reliable. But how is that technology [AI-driven lifestyle monitoring] installed in a home, and what does a patient do with it afterward? Those factors determine whether a product is reliable or not. Well, maybe not the product itself, but how it is used. For example, if a patient unplugs things, the product might be good, but its effectiveness is lost.”—*Healthcare technology expert *“It’s technology. If the prerequisites are met, the technology is reliable. AI is software-based, so when you input data, you get accurate models and predictions in return. As long as all connections are stable, the technology functions well. That’s been my experience. It’s like a chain of dominoes: if one piece fails, the entire process breaks down, including the technology. But this is true for any technology, even new ones.”—*ICT advisor
Negative experience with customer service	Negative experiences with the support provided by the supplier impact the successful use of AI-driven lifestyle monitoring systems.	*“It was a bit frustrating that at one point it just stopped working properly. And we did contact customer service a couple of times, we were also in contact with the mechanics, but each time they said: uh yes, try this or that, while actually it would have been nice if someone had just come by and fixed everything.”—*Informal caregiver
**3. Value proposition**	**The overall value of the technology.**	
Invasion of privacy	AI-driven lifestyle monitoring may be perceived as controlling and privacy-invasive.	*“It is, of course, an invasion of privacy, as you are being monitored remotely. This could be a potential barrier for the client themselves.”—*Nurse *“I believe that when someone needs a lot of help, their privacy is already significantly reduced because they constantly have people around, often different people. So, I think privacy and intensive care always go hand in hand.”—*Informal caregiver
Potential for overtreatment	The use of AI-driven lifestyle monitoring could lead to overtreatment as additional data are acted upon.	*“You end up tracking every detail of someone’s life. Even if I open the candy cabinet 36 times, that’s still my choice, my habit. Yes, the technology [AI-driven lifestyle monitoring] provides a lot of information, but I often wonder—do we really need to know all this? We seem to push everything to the extreme, continually adding treatments upon treatments.”—*Coordinator long-term homecare team
Strenuous feelings	Feelings like frustration, restlessness, and stress may arise from using the system.	*“It’s not very noticeable, but you can see it [the sensor], as it’s a small box […] that sticks out. This sometimes leads to confusion, like, ‘Am I supposed to do something with this? Should I press a button?’ We also noticed that some patients didn’t realize they needed to leave the plug in the socket, or they became restless by its presence.”—*Nurse *“But it can also have drawbacks. If there are constant notifications, you start to worry when there are no notifications, even if nothing is wrong, because it might just be a faulty sensor. So, yes, it can go both ways.”—*Coordinator long-term homecare team
Price	The high cost of AI-driven lifestyle monitoring potentially limits resources for other care.	*“It costs an incredible amount of money, and we’re already working with as few resources as possible. The system is very expensive and takes up a significant portion of the budget, which means I can provide less care elsewhere. Sometimes patients need more support that I can’t offer because we’re prioritizing the [AI-driven lifestyle monitoring] system. It’s a constant balancing act.”—*Coordinator long-term home care team
**4. Adopters**	**The adoption and continued use of the technology by different stakeholders.**	
Unclear actor upon notifications	The involvement of multiple teams and users creates confusion about who follows up on notifications and how this is communicated.	*“If this technology [AI-driven lifestyle monitoring] is going to be implemented, we need to ensure that there is enough staff to monitor and respond to alerts. It’s concerning to think about who will attend to a patient when something happens, is it the daughter or son, or is it us who need to do it?”—*Nurse *“You can’t always track everything. For example, if a notification is forwarded [to the external care centre], we don’t get any feedback. We see the original notification in our system and spend time trying to figure out what happened, but we only get a report in the file after someone from our team has handled it. This lack of feedback slows us down.”—*Coordinator long-term home care team
Training needed to use the system	Caregivers experience a lack of essential training to effectively use the system.	*“First, it’s helpful to understand the basics—how the sensors work, what they register, and why you use them. That helped us start on a positive note. Now, what I really need is support interpreting the information. It would be great to have someone with experience look at the data with us and help us understand: ‘What does this mean? What should we look for? What additional questions do we need to ask?’”—*Nurse
Change in job priority	Caregivers express fears of losing their jobs or needing to adapt their roles due to the introduction of AI-driven lifestyle monitoring systems.	*“That’s a huge threat, I think, for healthcare professionals. Many believe their jobs are at risk, which isn’t true at all. However, that message doesn’t always get through.”—*Director of home care
Notification overload	Caregivers are overwhelmed with information from AI-driven lifestyle monitoring systems.	*“What we see now is that the system generates many alarms, and these alarms don’t resolve themselves. It ends up causing stress because you’re constantly dealing with it. Instead of offering support, it often creates more work.”—*Coordinator of long-term home care team *“If we receive all the input, we would be overwhelmed with information, which is something we can’t handle. I think having a summary, for example, would be sufficient. I don’t need to know what she does every minute of the day. That’s not interesting to me. A general overview is much more important for us.”—*Informal caregiver
**5. Organization**	**The organization in which the technology has been implemented.**	
Lack of capacity	A lack of capacity to interpret data and act on notifications hinders the system’s effectiveness.	*“If you then consider […], during a shift, you repeatedly get a notification to respond to something you are almost 100 percent certain is a false alarm, but you still have to go there. That also affects very urgent care requests, where the client just has to wait longer. It is jeopardizing your on-call time.”—*Nurse
Behavioral change in technology use is needed	Behavioral change of caregivers is needed to successfully implement technologies like AI-driven lifestyle monitoring.	*“Especially because the technology [AI-driven lifestyle monitoring] is not yet the new normal, there is still a lot of work to be done. It truly requires a behavioural shift and a genuine acceptance of its added value.”—*Innovation manager *“You need to raise awareness and drive change among employees, who are mostly trained to provide direct care. There needs to be a shift in mindset: providing care should no longer be the first step, it should be the last. The focus should be on utilizing technology before delivering care.”—*Healthcare technology expert
Support deficiency	Formal caregivers experience a lack of support in using AI-driven lifestyle monitoring leading to frustration.	*“At the moment, it’s too complicated to request and install it, or that we have to do it all ourselves, then it doesn’t stand a chance of success.”—*Nurse
**6. Wider system**	**The wider regulatory and collaborative context around the current use of the technology.**	
Lack of reimbursement	A lack of clarity regarding financial reimbursement complicates implementing AI-driven lifestyle monitoring.	*“What I find very unfortunate is that there are no proper financing arrangements in this, at least in the healthcare side, and the market actually drives the price.”—*Innovation manager
Prevention costs and benefits are not aligned	AI-driven lifestyle monitoring creates costs within long-term care for older adults and benefits outside long-term care through prevention.	*“When you start focusing on prevention, and lifestyle monitoring is of course also largely used for prevention, preventing worse, you know. If that amount of care then becomes less with us, then it also delivers something for us. But it’s also very often the case that we actually prevent acute situations, a visit to the curative side of healthcare, and then that is where the main benefits lie.”—*Innovation manager
**7. Embedding and adaptation over time**	**The long-term integration and use of the technology.**	
–	–	

Next to these specific facilitators and barriers, participants also shared remarks related to working with technology in general, such as the need for open communication, a clear vision, colleague support, and varying motivation among users. However, as these aspects were not specifically relevant to the implementation of AI-driven lifestyle monitoring directly, these remarks are not included in the reporting of this paper.

### Facilitators

Several facilitators were mentioned regarding the implementation of AI-driven lifestyle monitoring. In particular, it was seen as versatile (domain *condition)*, unobtrusive (domain *technology*), offering clear values to all stakeholders (domain *value proposition*), and of continued regional and national interest (domain *wider system/embedding over time*). Most facilitators were found in both organizations and with all participant types.

#### Condition

People with cognitive decline were highlighted as a particularly suitable group, but AI-driven lifestyle monitoring could also be beneficial for other older adults. For example, lonely older adults, individuals with multiple sclerosis and with acquired brain injury, may also find value in it. People dealing with other somatic conditions such as walking difficulties, an increased risk of falling, and other age-related ailments could also benefit from its use by detecting changes in gait patterns and passively alerting caregivers if a fall occurs.

#### Technology

The desired functionality of AI-driven lifestyle monitoring includes a clear presentation of observations, minimal notifications (ideally only for deviations), and adaptability to changing user and patient needs, such as in cases of progressive disease. Participants expressed enthusiasm about the material and technical features of AI-driven lifestyle monitoring, particularly its ability to passively alert using a minimally privacy-invasive set of sensors. Furthermore, participants indicated the importance of a design that is unobtrusive to older adults, particularly those with dementia.

#### Value proposition

The core value of AI-driven lifestyle monitoring was seen to support safely living at home for longer. According to participants, it creates this value by collecting additional and objective data which are translated to tailored and timely interventions. It helps understanding older adults’ daily routines better, making it easier to identify changes early on and act accordingly, preventing escalation. Participants indicated that because AI-driven lifestyle monitoring is preventive and can streamline the process of assessing situations, it has the potential to reduce caregivers’ workload. In addition, AI-driven lifestyle monitoring was valued for providing reassurance to older adults as well as formal and informal caregivers. This reassurance results not only from its ability to issue alarms in urgent care situations but also from its AI-driven analysis of lifestyle changes, enabling the detection of deviations from normal patterns over extended periods. This reassurance seemed particularly valuable for informal caregivers, as they cannot always be physically present.

#### Adopters

AI-driven lifestyle monitoring is considered patient-aligned as it is seen being in line with the interests of the patient. This theme revolves around the notion that AI-driven lifestyle monitoring provides additional and objective data, enabling care to be customized to the older adults’ unique needs.

#### Wider system

Participants observed that government policies promote ageing in place, even with more complex care needs. Participants shared that older adults increasingly want to stay independent for as long as possible. They believe this is not just encouraged by the government but is also valued more by society. In this shift, AI-driven lifestyle monitoring can help support and promote independent living. From this shared value, formal caregivers and management also see potential in establishing more comprehensive national, regional, and neighborhood agreements to support collaboration in using AI-driven lifestyle monitoring. This was particularly true for participants who already had experience with AI-driven lifestyle monitoring. For example, they suggested that responding to notifications could involve a neighbor instead of a formal caregiver, enhancing the system’s usability and effectiveness. Additionally, they indicated that sharing data with other organizations could be valuable for addressing issues that extend beyond the care organization. Lastly, there were suggestions that responsibility for implementing AI-driven lifestyle monitoring could regionally be shared, with municipalities playing a role alongside healthcare organizations.

#### Embedding and adaptation over time

Participants believe that AI-driven lifestyle monitoring will become increasingly useful in the future, particularly as the number of older adults with dementia continues to grow, resulting in more complex care needs. They expect AI-driven lifestyle monitoring to play an increasingly critical role in addressing these challenges, with rapid advancements in care technology on the horizon. Collaboration among different organizations is highlighted as a vital factor for the future of healthcare. As the perceived necessity of AI-driven lifestyle monitoring increases, participants also anticipated greater adoption of these systems and more collaboration between formal and informal caregiver in using it.

### Barriers

Participants noted several barriers regarding the implementation of AI-driven lifestyle monitoring. Barriers were perceived mainly in the organizational, adopters and surprisingly, also the value proposition domain. Participants viewed AI-driven lifestyle monitoring as a costly, stressful invasion of patients’ privacy (domain *value proposition*), for which there was little capacity and organizational support (domain *organization*), that added to work pressure due to notification overload (domain *adopters*). Additionally, barriers were more likely reported by participants employed in the organization that already had experience using AI-driven lifestyle monitoring. These participants highlighted issues like negative experiences with customer service (domain *technology*), high costs of the system (domain *value proposition*) and lack of support, capacity, and training (domain *organization/adopters*).

#### Condition

Regarding the condition it was noted that AI-driven lifestyle monitoring may be less suitable in certain situations. For example, some patients may be distrustful or suspicious, possibly due to a lack of understanding of their illness. Additionally, for individuals who are highly restless, the use of AI-driven lifestyle monitoring could potentially worsen their restlessness or anxiety.

#### Technology

Participants who had experience using AI-driven lifestyle monitoring shared mixed experiences about the system’s reliability, indicating challenges in determining whether AI-driven lifestyle monitoring is accurately measuring what it is intended to measure and possible limitations of the system such as its ability to display a patient’s location within the home and its functionality being restricted to single households. They felt these aspects needed further development to better suit their patients’ needs. Some participants also reported negative experiences with system errors, leading to doubts about the system’s reliability which reportedly could lead to compromising patient safety. Next to that, participants criticized the customer service provided by the supplier of the AI-driven lifestyle monitoring system, highlighting the need for more practical support in case of (system) errors.

#### Value proposition

Some participants believe AI-driven lifestyle monitoring can feel intrusive, especially when it comes to balancing the privacy of older adults with its use, particularly for vulnerable individuals like persons with dementia. However, it was also noted that privacy concerns should not be exaggerated, provided there is clear communication with the patient and their family. Some participants pointed out that older adults are often already familiar with compromising some degree of privacy, for instance, when they live in a nursing home. Another concern noted was the potential for overtreatment of patients following the implementation of AI-driven lifestyle monitoring as additional data gathered through the AI-driven analysis of lifestyle changes is now acted upon.

#### Adopters

AI-driven lifestyle monitoring is used by various disciplines, including nurses from an external care center, nighttime and long-term home care nurses, dementia case managers, and informal caregivers. Because of the many disciplines involved, interdisciplinary collaboration between all stakeholders is essential. Yet, participants shared that limited communication between disciplines led to frustration and stress for everyone involved. There is a need for clear accountability regarding notifications, ensuring both formal and informal caregivers understand who is responsible for responding to specific notifications. In order to do so, both formal and informal caregivers highlighted the need for more training on interpreting and using the system’s data, as they find this aspect challenging. Additionally, concerns have been raised about the excessive amount of information and notifications generated by AI-driven lifestyle monitoring, which could place a significant burden on (in)formal caregivers.

#### Organization

Formal caregivers believe that while the limited resource capacity is sufficient to implement the AI-driven lifestyle monitoring system (e.g., decision-making, installation, and setup), there is insufficient time to incorporate its use into their daily activities. This limits their ability to effectively interpret the data and respond to the system’s notifications. They also report facing high work pressure, making them hesitant to take on additional responsibilities, such as integrating new technologies. The extra burdens associated with using the system, including the need for interdisciplinary collaboration, difficulties in understanding and interpreting data, and system errors, have increased their perceived workload. However, participants also acknowledged the potential of the system to reduce this pressure in the long term.

Participants had varying opinions on who should be responsible for adopting the AI-driven lifestyle monitoring system. Informal caregivers believed that formal caregivers should take the lead in adopting AI-driven lifestyle monitoring. However, formal caregivers reported feeling unsupported by the management team in using AI-driven lifestyle monitoring, which led to frustration. Management, on their end, indicated that a change in behavior regarding working with technology among both formal and informal caregivers is necessary for the successful implementation of technologies like AI-driven lifestyle monitoring. To address this situation, it was suggested that a specialized team should be established to manage the implementation, provide technical assistance, and answer questions. This would streamline the process and make the system easier to use from a caregiver’s perspective. Providing training, education, and ongoing updates for healthcare professionals and informal caregivers were also considered important in ensuring successful implementation.

#### Wider system

Finally, participants have expressed concerns about unclear reimbursement agreements after the initial pilot phases in the organization that had used AI-driven lifestyle monitoring. Relying on organizational innovation budgets is not a sustainable long-term solution, partly due to the high cost of the system and the lack of national reimbursement agreements. Future reimbursement depends on the system being recognized as an evidence-based technology, which could lead to more health insurance companies covering it. Participants indicated that these agreements are crucial not only to finance the system itself but also to support the costs of user training and system implementation. Another financial aspect lies in the fact that AI-driven lifestyle monitoring is often used for prevention, where the costs and benefits affect different domains. For example, participants said that the care organization pays for the system, but the financial benefits, such as avoiding urgent care, mostly go to another domain (cure organizations such as hospitals).

## Discussion

The aim of this study was to identify the facilitators and barriers that formal and informal caregivers as well as management perceive regarding the implementation of AI-driven lifestyle monitoring systems in long-term care for older adults. The study revealed that AI-driven lifestyle monitoring is perceived as adaptable to various conditions, unobtrusive, and clearly beneficial to stakeholders. It is also seen as having ongoing regional and national relevance, among others because of governmental policies promoting aging in place. However, it is also viewed as an expensive and stressful intrusion into patients’ privacy, with limited organizational support. Additionally, the technology contributes to increased work pressure due to an overload of notifications.

To provide a comprehensive overview of barriers and facilitators, the study includes perspectives from both a healthcare organization that has adopted AI-driven lifestyle monitoring and one that has not. While most themes were found in both organizations, there are notable differences. One aspect that stands out in this regard is that 9 out of 16 barriers were only reported by (informal) caregivers and management of the organization that had adopted AI-driven lifestyle monitoring, but not by those we did not have experience with such a system yet. This could potentially indicate an AI hype, in which overly positive expectations of AI in healthcare, may ultimately result in unrealistic assumptions about its potential and impact (Strange, 2024). This optimistic viewpoint was also present when participants discussed embedding of AI over time. No barriers were identified in this domain, and participants believed that AI-driven lifestyle monitoring will only become more valuable as the aging population increases, leading to more complex care needs. However, it could also be argued that some of the barriers are system-specific factors unique to this specific organization and technology, such as customer service experiences, as well as practical issues like increased work pressure due to notification overload, which require experience to be fully recognized. One of these aspects specifically recognized in the adopter organization was the need for additional training for both formal and informal caregivers. This aligns with research highlighting its impact on healthcare professionals’ ability to make informed decisions (Shaik et al., 2023) and highlights the necessity of including AI-training in educational curricula (Labrague et al., 2023; Taneri, 2020) for which the needed competencies of nurses to work with AI-driven lifestyle monitoring are researched (Groeneveld et al., 2025).

Furthermore, this study found that the unobtrusive nature of AI-driven lifestyle monitoring is a positive factor facilitating its use. This aligns with previous research, which recognized unobtrusive in-home monitoring as a valuable tool for monitoring older adults (Sharma et al., 2023; Wrede et al., 2021), regardless whether it is AI-driven or not. Like their findings, our study also highlighted benefits such as prevention, reassurance, and personalized care, while noting concerns about privacy and information overload. Similarly, a recent study by Tian et al. (2024) emphasized that continuous monitoring of daily activities helps healthcare professionals understand routines and health status, enabling personalized care and supporting the independence of older adults (Tian et al., 2024). It was also indicated that the technology could be more widely used if it was financed or reimbursed, a notion also found in our results.

Some participants raised concerns about the privacy of older adults, particularly those who are vulnerable, such as individuals with dementia. This concern is mirrored in other studies, which highlight privacy and data security concerns as critical factors influencing the willingness to integrate AI-driven technologies into care (Tran et al., 2019). However, other participants noted that older adults requiring intensive assistance often experience reduced privacy due to the constant presence of caregivers, such as when living in a nursing home. Additionally, privacy and safety may not always align regardless of whether data collected is AI-driven or not, a point also highlighted in an earlier study where informal caregivers expressed a willingness to sacrifice some privacy in order to enhance patient safety (Zwierenberg et al., 2018). Another barrier identified is the potential for overtreatment, as AI-driven lifestyle monitoring may generate additional health insights that prompt unnecessary interventions. This aligns with previous research highlighting how an emphasis on prolonging life can lead to overtreatment at the expense of quality of life (Cruz et al., 2022). To mitigate overtreatment, it is suggested that healthcare professionals should engage in open discussions with older adults and families about the intervention’s risks and benefits and regularly review treatment to ensure it aligns with the older adults needs (Ooi, 2020).

Participants in our study had differing views on who should lead the implementation of AI-driven lifestyle monitoring. Informal caregivers felt that formal caregivers should take the initiative, while formal caregivers expressed frustration due to a lack of support from management in using the technology. Management, in turn, emphasized the need for a behavioral shift among both formal and informal caregivers regarding working with technology to ensure the successful integration of such technologies. This status quo means that all involved stakeholders will look at each other hindering successful implementation of AI-driven lifestyle monitoring. It could be suggested that creating a shared vision on the use and role of AI-driven lifestyle monitoring can drive the implementation by bridging the different views of involved stakeholders. Another concern related to multi-stakeholder collaboration revolves around the shared responsibility towards the number of notifications produced by AI-driven lifestyle monitoring. Participants expressed concerns about the volume of notifications generated by the technology: there is a fear of notification overload, which could overwhelm both formal and informal caregivers. This is consistent with findings from another study into home-based dementia care, which noted that such overload can impose an additional burden, making caregivers’ work less predictable and disrupting their routines (Wrede et al., 2021). Furthermore, it was noted that there was uncertainty regarding who should respond to such notifications. Participants mentioned that with so many individuals involved, it was unclear who should take action. This uncertainty of responsibilities was also found in another study focusing on (non-AI-driven) lifestyle monitoring using infrared sensors to record movements (Braspenning et al., 2022). This situation could be explained by a phenomenon commonly described within psychology known as the bystander effect (Siligato et al., 2024): when other people are around, an individual is less likely to take action. In our study, participants expressed concerns that because of this, perhaps no one would step in to help and questioned what would happen in that situation.

In the wider system domain, it was recognized that national regulations and priorities, such as aging-in-place policies, will potentially support the use of the system. However, there was limited attention to international developments in this wider system that could significantly influence implementation. For instance, the introduction of international legislation like the EU AI Act (*EU AI Act: first regulation on artificial intelligence*, 2023) may have a direct impact on the legal and ethical requirements for AI systems such as aspects like responsible, validated, and equitable data exchange between states.

Although some comments were made about the need to prevent a reduction in human contact, the concept of dehumanization linked to AI in healthcare was not directly identified as a theme in our study, despite being highlighted in other research (Rubeis, 2021). Dehumanization refers to the reduction of a person to mere data or a set of characteristics, often undermining the individual’s dignity and humanity. A possible explanation for its absence in this study’s findings could be the specific focus of the AI technology we examined, which is designed to enhance understanding of the patients being cared for. This is evident in the tailored care theme that was identified, revolving around the notion that AI-driven lifestyle monitoring enables care to be customized to the patient’s unique needs. In this context, the AI system contributes to care tailored to actual patient needs by collecting additional and objective data, leading to a deeper understanding of the patient rather than dehumanizing them by separating the patient from their data.

### Recommendations for implementation

The findings of this study provide learnings for long-term care organizations wanting to implement AI-driven lifestyle monitoring systems or other AI-based technologies. To facilitate successful implementation, we recommend five actions:

- *Starting a dialogue on privacy concerns*: given the privacy concerns identified in this study, it is essential to engage in open dialogue with (future) users regarding data security in long-term care for older adults. Expectations should be exchanged with older adults and informal caregivers regarding the extent to which the benefits of reassurance outweigh the loss of privacy.
- *Providing training*: effective training on both the use and interpretation of AI-generated data is important for successful implementation. Additionally, attention should be given to maintaining the human aspect of care, clarifying responsibilities for acting on system notifications and on potential risks of overtreatment.
- *Create a shared vision on the role and use of AI in care delivery*: to facilitate successful implementation, organizations should establish a shared vision between all parties involved on the role and use of AI in care delivery; appointing a dedicated implementation team or involving opinion leaders and clinical champions can help drive adoption (Miller et al., 2021).
- *Take part in (re)design activities*: healthcare organizations should take an active role in the (re)design of AI-driven lifestyle monitoring systems. Practical experiences, including issues such as notification overload, system reliability, and customer service, should be used into system (re)design to ensure usability and effectiveness in real-world settings.
- *Consider the financial aspects*: discussions should focus on the financial feasibility of AI-driven lifestyle monitoring and explore ways to fairly distribute financial benefits among stakeholders. This approach supports preventive care, helping to reduce the need for urgent interventions and the costs that come with them.

### Strengths and limitations

A key strength of this study is the inclusion of a diverse range of stakeholders, including not only formal caregivers but also informal caregivers and management (among others supporting staff), involved in healthcare technology implementation. This multidimensional perspective, including both an adopter and nonadopter organization, ensures a broad understanding of context, addressing all relevant aspects of AI-driven lifestyle monitoring implementation. Another strength lies in the consistent application of the NASSS framework (Greenhalgh et al., 2017), which structured both data collection and analysis, increasing the credibility, dependability, and conformability of the findings. The position of two of the researchers within the organization provided valuable contextual knowledge, enhancing the analysis by providing a deeper and more accurate interpretation of the data. However, it also introduces potential limitations, as participants may have felt the need to give socially desirable responses, and the researchers’ perspective could have influenced the thematic construction of the findings. To minimize this risk, two other researchers not involved with the studied organizations reviewed and refined the data analysis. During one of the interviews with an informal caregiver, one patient was present and occasionally contributed. As patients were not the primary focus of this study, the caregiver’s insights remained central, and the interview was retained for its relevance to the research objectives. Finally, there is also the potential for selection bias, as participants were recruited based on their involvement in healthcare technology and their willingness to participate. However, by using both purposive sampling (based on organizational charts and job descriptions) and snowball sampling (Parker & Geddes, 2019), this risk for selection bias was minimized.

## Conclusion

This study identified the facilitators and barriers to the implementation of AI-driven lifestyle monitoring in long-term care for older adults, as perceived by formal and informal caregivers, as well as management in organizations that have and have not adopted this novel technology. The findings highlight the potential of AI-driven lifestyle monitoring as a versatile, patient-centered, and unobtrusive technology that can enhance tailored care for older adults, provide reassurance for (in)formal caregivers, and improve care efficiency within healthcare organizations. However, successful implementation requires addressing several challenges, including privacy concerns, the potential for overtreatment, system reliability, training needs, notification overload, organizational alignment, and ambiguity regarding responsibility for acting on notifications. Additionally, broader contextual factors such as national regulations, strategic partnerships, and financial considerations further influence the outcomes of implementation. This study offers recommendations to optimize implementation strategies, making sure that AI-driven lifestyle monitoring can be effectively integrated into long-term care for older adults. The insights gained contribute to a broader understanding of how AI technologies can be successfully adopted in similar healthcare settings. Ultimately, a comprehensive approach that considers individual, organizational, and societal levels is essential for making use of the potential of AI-driven lifestyle monitoring in long-term care for older adults.

## Data Availability

The data are not publicly available due to privacy concerns and confidentiality agreements. For inquiries, please contact the corresponding author. This study was not preregistered.
